# Practical Observations on the Teeth, Designed for Popular Use

**Published:** 1852-10

**Authors:** 


### BIBLIOGRAPHICAL.

*Practical Observations on the Teeth, designed for Popular Use.* By Henry Jordan, Dentist, Derby. London, Samuel Highley, 32 Fleet street, 1851, pp. 104.

This neat little book has been received with pleasure. It is well written and seems adapted to diffuse correct elementary knowledge on dental matters among the public, who, with all their intelligence, are yet patiently submitting to the manipulations of *soi disant* dentists, as ignorant as themselves of the physiology, pathology and therapeutics of the teeth.

